# Supplementary material for: Identification of photoperiod-induced specific miRNAs in the adrenal glands of Sunite sheep (Ovis aries)
Source: Front Vet Sci. 2022 Jul 22;9:888207. doi: 10.3389/fvets.2022.888207 (PMC9354845; doi:10.3389/fvets.2022.888207)
Supplement: Supplementary file 1 [file Data_Sheet_1.ZIP › Supplementary file/miRDeep2 file.zip!/miRDeep2 file/Know_Structure/SPLP42A3/oar-mir-103.pdf]

miRBase precursor	: oar-mir-103
Total read count	: 9862
oar-miR-103 read count	: 9861
remaining reads	: 1

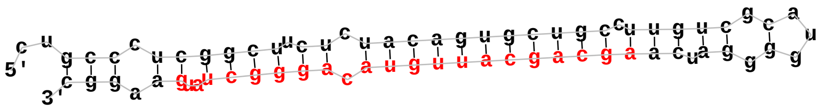

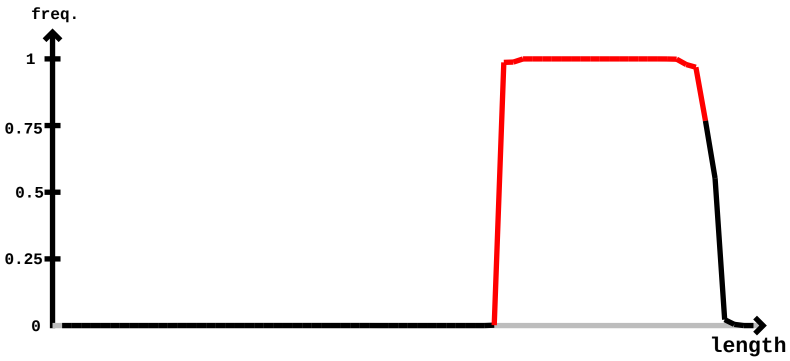


```
                                                                      oar-miR-103
5'- cugcccucggcuucucuacagugcugccuugucgcauggggaucaagcagcauuguacagggcuaugaaggc   -3'    exp
    ..(((.((((((.((.(((((((((((.(((((.(...).)).))))))))))))))).))))))..)).)))    reads      mm         sample
    ...........uucucuacagugcugccuugu........................................    1          0          seq
    ............................................aagcagcauuguacagggcuaug.....    7          0          seq
    ............................................aagcagcauuguacagggcuauga....    8          0          seq
    .............................................agcagcauuguacaggg..........    2          0          seq
    .............................................agcagcauuguacagggc.........    9          0          seq
    .............................................agcagcauuguacagggcu........    197        0          seq
    .............................................agcagcauuguacagggcua.......    101        0          seq
    .............................................agcagcauuguacagggcuau......    1976       0          seq
    .............................................agcagcauuguacagggcuaug.....    2090       0          seq
    .............................................agcagcauuguacagggcuauga....    5142       0          seq
    .............................................agcagcauuguacagggcuaugaa...    165        0          seq
    .............................................agcagcauuguacagggcuaugaaA..    34         1          seq
    .............................................agcagcauuguacagggcuaugaag..    1          0          seq
    ..............................................gcagcauuguacagggcuau......    1          0          seq
    ..............................................gcagcauuguacagggcuaug.....    4          0          seq
    ..............................................gcagcauuguacagggcuauga....    8          0          seq
    ...............................................cagcauuguacagggcuau......    4          0          seq
    ...............................................cagcauuguacagggcuaug.....    21         0          seq
    ...............................................cagcauuguacagggcuauga....    72         0          seq
    ...............................................cagcauuguacagggcuaugaa...    9          0          seq
    ...............................................cagcauuguacagggcuaugaaA..    9          1          seq
    ................................................agcauuguacagggcuaugaa...    1          0          seq
```
